# Cleanliness

**Published:** 1878-04

**Authors:** 


### CLEANLINESS

Of person—the strictest cleanliness—should be among the earliest and most imperative of our teachings to our children; not external cleanliness, but that which is most promotive of health—cleanliness of the skin and the garments which are nearest to it. With what contempt would we look on the best dressed and handsomest person on the street, if we could know that the feet had not been washed for a week, nor the inner garments for a month; and yet it is undeniable that many persons are satisfied that the outer garment should be unexceptionably clean; if that be whole and without a rent, it matters not how soiled and tattered those out of sight are. No such mind can be pure; it implies a deceptiousness of heart which it is impossible to admire. Let mothers especially charge it upon their daughters from earliest life, that it is actually as discreditable to have a hole in the stocking as in the silk dress; that a splotch or stain, or grease spot on an inner garment, is not less unpardonable than if found on a shawl or cloak, or bonnet. Let every mother feel that cleanliness, temperance and thrift, are the antipodes of filth, bestiality and improvidence, and that spotless cleanliness of person, and purity of mind, are absolutely inseparable.

